# Solitonic conduction of electrotonic signals in neuronal branchlets with polarized microstructure

**DOI:** 10.1038/s41598-017-01849-3

**Published:** 2017-05-31

**Authors:** R. R. Poznanski, L. A. Cacha, Y. M. S. Al-Wesabi, J. Ali, M. Bahadoran, P. P. Yupapin, J. Yunus

**Affiliations:** 1 Faculty of Biosciences and Medical Engineering, Universiti Teknologi Malaysia, 81310 Johor Bahru, Johor Malaysia; 2 Laser Centre, IBNU SINA ISIR, Universiti Teknologi Malaysia, 81310 Johor Bahru, Johor Malaysia; 3 Faculty of Science, Universiti Teknologi Malaysia, 81310 Johor Bahru, Johor Malaysia; 4 Department of Physics, Shiraz University of Technology, Shiraz, 313-71555 Iran; 5 Department for Management of Science and Technology Development, Ton Duc Thang University, Ho Chi Minh City, District 7 Vietnam; 6 Faculty of Electrical & Electronics Engineering, Ton Duc Thang University, Ho Chi Minh City, District 7 Vietnam

## Abstract

A model of solitonic conduction in neuronal branchlets with microstructure is presented. The application of cable theory to neurons with microstructure results in a nonlinear cable equation that is solved using a direct method to obtain analytical approximations of traveling wave solutions. It is shown that a linear superposition of two oppositely directed traveling waves demonstrate solitonic interaction: colliding waves can penetrate through each other, and continue fully intact as the exact pulses that entered the collision. These findings indicate that microstructure when polarized can sustain solitary waves that propagate at a constant velocity without attenuation or distortion in the absence of synaptic transmission. Solitonic conduction in a neuronal branchlet arising from polarizability of its microstructure is a novel signaling mode of electrotonic signals in thin processes (<0.5 μm diameter).

## Introduction

### Motivation

The dynamic structure of electrical activity in the nervous system is information-rich^1^ with action potentials considered by most neuroscientists to form the basis of electrical signaling^2^. Action potentials are electrical spikes quantitatively described through a system of nonlinear equations developed by Alan Hodgkin and Andrew Huxley^3^ (hereafter H-H). The H-H model treats the nerve as an electrical cable upon which a propagating action potential is driven by dynamics. It is inscribed in a porous medium where currents leak through resistors and the membrane is a dielectric represented by a classical capacitor. Remarkably, the H-H model provides a quantitatively accurate description of electrical phenomena in neurons through properties of solitary waves. In particular, the nerve impulse, which is a solitary wave, is localized and remains stable, but destabilizes after collision with other action potentials^4^. Indeed, this has been understood as a golden rule of cable theory for neurons without microstructure^5^.

Conferring with the H-H model, colliding action potentials will annihilate due to the in-activation of the sodium conductance (the refractory period). However, there are also studies with the findings that two colliding action potentials will not lead to their mutual annihilation right away. Instead, they reflect from one another and eventually they annihilate, thereby projecting solitonic properties, see refs 6, 7. Nevertheless, action potentials are solitary pulses and not solitons since they do not preserve their shape and velocity after collision. In other words, the crucial test for solitary pulses to be solitons is robustness to collision.

The early work of Aizawa and colleagues^8^ proved the existence of solitons in dissipative media ﻿referred to as ‘dissipative solitons’, which are dubbed quasi-solitons as dissipation is evident after collision. Although quasi-solitons (or soliton-like) behavior has been observed in many excitable systems^9^, the existence of solitons in reaction-diffusion systems was first shown by Tuckwell^10, 11^. A reaction-diffusion system describes the concentration of charged ions which is mathematically equivalent to the conduction of current as flow of electrical charges in an electrical cable model. For example, a hybrid model developed by Meier and colleagues^12^ proposed a voltage-dependent Nernst potential in a reaction-diffusion system where the membrane potential exhibited solitonic properties on an assumption that H-H model behaved like an electrochemical, reaction-diffusion system.

The H-H model excludes microstructure and simply assumes a homogenized resistive fluid for the interior of the giant axon of *Loligo*. Earlier cable modeling efforts treated the intracellular medium of neurons to be a homogeneous resistive fluid of 70 Ωcm as measured for electrolyte solution only, see ref. 13. Meier and colleagues^12^ observed in neurons with radii several orders of magnitude smaller than that of the giant axon of *Loligo*, to have significant intracellular charge depletion. For these reasons, a cable model is required that specifically addresses electrotonic signal propagation in small neuronal processes with microstructure^14, 15^. Modeling efforts by Shemer and colleagues^16^ have explicitly considered endoplasmic reticulum encased within a core-conductor. However, such cytoplasmic inhomogeneity in the microstructure does not explicitly take into consideration intracellular capacitive effects due to charge flow of molecular ions as a result of protein polarization^17^ or include capacitive effects only in the extracellular space^18^.

A cable model with passive membrane that includes the diffusion of free charge was derived in which it was confirmed that the diffusion of free charge contributes through the conduction of current flow arising from capacitive charge-equalization and axial capacitive effects^19^. The model was further developed by including polarization current due to dispersion of bound charge on the surface of endogenous structures in the cytoplasm^20^. This resulted in polarization current arising from electrostatic interactions between charges/dipoles in the cytoplasm at slow varying electric fields (e.g., quasi-electrostatic conditions) causing self-excitability with a variety of different electrical signaling patterns created by charges held by the microstructure.

Gonzalez-Perez and his colleagues^21^ had shown experimentally how a collision between two electrical pulses did not result in their annihilation. This contrasts to the collective idea of annihilation due to the existence of a refractory period in the H-H model^4, 5^, implying that active conduction is not universal for all types of nerve fibers after all. They proposed that their results can be explained in terms of a soliton model^22, 23^ based on the assumption that changes in lateral density of the membrane are proportional to changes in voltage. And mechanical signals propagate in phase with electrical pulses^24^, so that a thermodynamic theory of a nerve impulse could be seen as complementary, which is nonelectrical and consistent with an adiabatic wave (e.g. sound wave), see ref. 25. Mechanical changes during an action potential can produce a self-sustaining and localized density pulse propagating over long distances without loss of energy with minimum propagation speed of 100 m/s, see ref. 26. However, Gonzalez-Perez and his colleagues^21^ experimental result have shown electrical pulses (which they presumed to be action potentials) of less than 10 mV in axons with diameter of lateral giant (*Lumbricus terrestris*) axons between 4 to 100 μm with velocities well under 10 m/s. Therefore their results are not compatible with a mechanical soliton model. In this paper, we introduce an alternative signaling mechanism based on solitonic conduction of electrotonic potentials that is compatible with their experimental results.

## Methods

### Intracellular capacitive effect of microstructure

Microstructure contains a dense meshwork of cytoskeletal structures made up of neutrally charged macromolecules assembled from amino-acids using information encoded in genes in a polypeptide chain linked by peptide bonds. At random orientations, the arrangement of charges in some macromolecules are static when no electric field is present, but in the presence of an electric field, macromolecules become polarized and separate by orienting the dipole moments of polar molecules resulting in the formation of permanent dipoles which attract surface charge densities (so-called bound charge densities) causing a displacement of charge. These bounded charges produce intracellular capacitive effects that can contribute to ionic current flow arising from the dispersion (fluctuation) of bound charge by affecting the voltage created by charge flow of molecular ions in the intracellular fluid.

**Figure 1:**
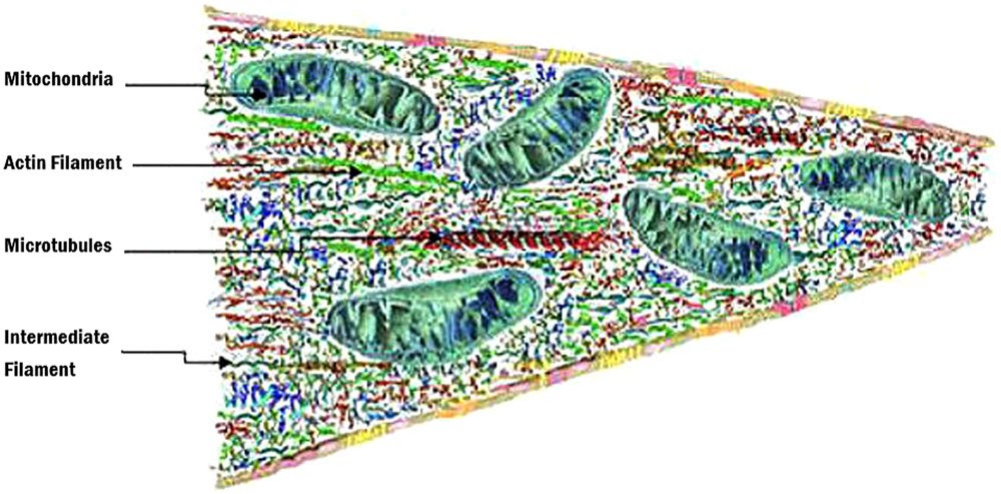
A schematic illustration of a longitudinal section of the neuronal branchlet with microstructure showing cytoskeletal structures (interlinking actin filaments, intermediate filaments, and microtubules) and closely compressed accumulated membranous organelles that extend to distal part of a branchlet. The cytoskeleton is a network of connected actin and intermediate filaments, and microtubules including mitochondria in neurons causing a gradual tapering towards one end. The microstructure constitutes a fissured domain of the cytosol (fluid that contains organelles, comprising the cytoplasm). The mitochondrial membrane is the largest organelle (∼0.2 μm) within the microstructure and dominates the constituency of the cytoskeletal structures since endoplasmic reticulum does not enter into branchlets below a micron.

One way to increase the densities of electrical charges within a neuronal branchlet (i.e., thin dendrite or thin axon that is under half a micron in diameter) is to polarize microstructure in the presence of quasistatic electric field (see Fig. 1). For example, the flux generated by electrical conduction of a polarized current in the longitudinal direction (along the cable length) arising from polarizability of its microstructure. In Fig. 1 the branchlet is shown to be tapering, while the model to be derived is intended to represent only for nontapered cylindrical representation.

The cytosol (fluid that contains orgenelles, comprising the cytoplasm) is a porous medium of the intracellular fluid assumed to be homogeneous and purely conducting, i.e., the electrical behavior of the medium is entirely characterized by a homogeneous electrical conductivity representing ionic homogeneity for all macromolecules. This is a simplification that suffices for the conduction process, focusing on homogeneous dielectric polarization^20^ without exploring the physicochemical nature of excitation. Consequently in the context of conduction process, ionic polarizability of individual molecules is not taken into account, but instead a continuum of molecular ions with macroscopic charge densities are used as a phenomenological description of the electrostatic interactions and transfer of information due to the interactions among the molecular ions in a fluid environment within neuronal branchlets.

### Cable model with homogenous microstructure

A homogenous microstructure is represented electrically in terms of intracellular capacitive effects that contribute to dielectric absorption, and nonlinear (voltage-dependent) transfer of macroscopic charge densities leading to inherent waveforms of electrical depolarization. Phenomenological description of quasi-electrostatic interactions of macroscopic charge densities held by the microstructure are modeled as flux generated by electrical contribution of polarization current in a homogenous core- conductor representation of a neuronal branchlet, assuming extracellular isopotentiality, spatial and ionic homogeneity and negligence of currents due to ionic concentration gradients among molecular ions in an electrolytic microenvironment.

**Figure 2:**
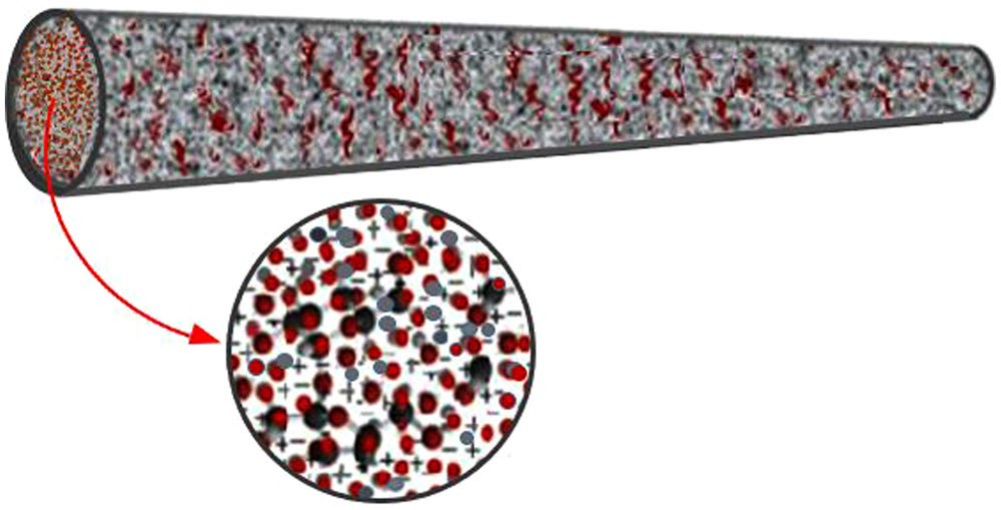
A schematic illustration of ionic inhomogeneity in a porous medium of a neuronal branchlet. The microstructure is indicative of macromolecules in an electrolyte solution (excluding subcellular membranes of mitochondria). The inset shows molecular ions in an electrolytic microenvironment within the neuronal branchlet.

Cable models of electrical activity in neurons assume a one-dimensional description of neuron geometry for describing the process of conduction^5^. In our cable model, we consider the microstructure to be a homogeneous dielectric of constant conductivity and permittivity in space and time and isotropic (same in x/y directions). We ignore anisotropic electrical properties, compare with refs 27, 28, assume the polarization current is restricted longitudinally along the cable length, and extracellular isopotentiality and quasi-electrostatic conditions prevail. Therefore in the context of conduction process, ionic polarizability of individual macromolecules is not taken into account, but rather macroscopic charge densities are used as a phenomenological description of the electrostatic interactions between charges/dipoles held by the microstructure (i.e., charge ‘soakage’). Our model includes a homogenous microstructure of a cable representation of a branchlet as an approximation to an inhomogeneous microstructure of an electrolytic cable representation of a branchlet (see Fig. 2).

### Basic equation of the model

**Figure 3:**
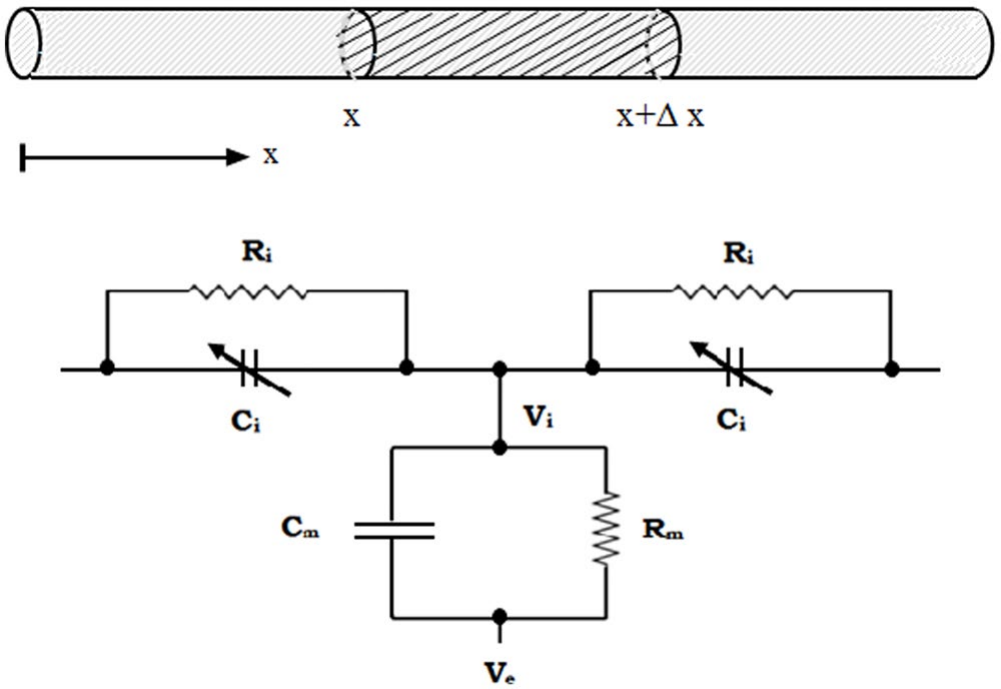
A segment of a neuronal branchlet as a cylindrical cable that supports the formation and propagation of electrical solitons reflective of the movement of macroscopic charge densities arising from polarizability of the microstructure, whose capacitance changes with voltage along a cable. The description of the ‘macroscopic’ electric parameters as a representation of a line charge in the branchlet caused by quasi-electrostatic interactions consists of infinitely long, infinitely thin distribution of charges uniformly distributed and denoted as charge per unit length of cable Q(x, t) (C/cm). The length increment Δx is shown where arrow indicates the convention that positive charge is in the direction of increasing x, which is the physical distance along the cable. It is assumed the cable is a homogeneous conductor with radial currents ignored. Below is an equivalent series-parallel RC circuit representing a patch of passive membrane in series with the intracellular medium represented by a voltage-dependent longitudinal (axial) capacitance C_i_(V) = C_i_ C(V_i_) of the cable (F/cm) in parallel with the intracellular resistivity (R_i_) of the cable (Ωcm). Definitions of electrical terms are: R_m_ = 2πrr_m_ is the membrane resistivity or resistance across a unit area of passive membrane (Ωcm^2^), C_m_ = c_m_/2πr is the membrane capacitance per unit area of membrane (F/cm^2^), C_i_ = c_i_/πr^2^ is the voltage-independent longitudinal capacitance (intracellular capacitance) per unit length of cable (F/cm), and c_i_ is the axial capacitance across unit length c_i_ = 2 ε_o_ πr^2^ (Fcm). See text for other definitions of electrical terms.

The membrane potential distribution can be derived from conservation of electric charge in a volume of cylindrical cable over a differential distance Δx as shown in Fig. 3 (top); see ref. 29. The cable has a radius (r), cross-sectional area (πr^2^) and perimeter around the membrane (2πr). If the conductivity of the extracellular medium is high, leaving the extracellular medium isopotential (i.e., V_e_ = 0) then the effect of the external potential on membrane potential (i.e., V = V_i_ − V_e_) is negligible.

If the membrane potential (V) in a neuronal branchlet is equal to the intracellular potential (V_i_) together with passive membrane as shown in Fig. 3 then the nonlinear cable equation with polarized microstructure can be written as ref. 20:

1 $${\rm{V}}+\partial {\rm{V}}/\partial {\rm{T}}={\partial }^{2}{\rm{V}}/\partial {{\rm{X}}}^{2}+{\rm{\gamma }}\,{\partial }^{3}{\rm{V}}/\partial {\rm{T}}\partial {{\rm{X}}}^{2}+{\rm{\kappa }}\,\partial \{{\rm{C}}({\rm{V}}){\rm{V}}\}/\partial {\rm{T}}$$

where V is the membrane potential (mV), γ = τ_ρ_/τ_m_ ≪ 1 and κ = γ (λ^2^/πr^2^) are both positive constants (dimensionless), τ_p_ = 2ε_r_ε_o_/σ < 1msec (Maxwell’s time constant), ε_r_ = 81 is the relative permittivity of sea water (dimensionless), ε_o_ = 7 × 10^—12^ F/cm is the fluid permittivity, τ_m_ = c_m_r_m_ (passive membrane time-constant in msec), λ = √(r_m_/r_i_) (electrotonic space-constant in cm), dimensionless time T = t/τ_m_ and dimensionless space X = x/λ, r_i_ is the core-resistance (or intracellular resistance) per unit length r_i_ = 1/(πr^2^σ) (Ω/cm), r_m_ is the membrane resistance across a unit length of passive membrane cylinder (Ωcm), c_m_ is the membrane capacitance per unit length of cylinder (F/cm). Note the core-resistance (or intracellular resistance) per unit length differs slightly from the intracellular resistivity R_i_ = 1/(2σ) (Ωcm) or volume resistivity of the intracellular medium, also referred to as specific resistance (1/σ) where σ is the electrical conductivity (S/cm).

Equation (1) contains two additional terms in the classical cable equation; see refs 5, 30, 31, which reflect the intracellular capacitive effects due to polarization current. A physical interpretation of these new terms requires quantitative verification that is lacking even when γ is small and hence κ is small, yet the intracellular capacitive effects do form a significant dielectric, and not simple passive RC filter properties. The first new term γ ∂^3^V/∂T∂X^2^ is a linear dissipative term due to surface-charge equalization between polarized intracellular free charges on endogenous structures within the microstructure^19^. The other new term ∂{C(V)V}/∂T is the charge ‘soakage’ due to polarization current within the microstructure produced by the bounded charges on endogenous structures^20^. In a neuronal branchlet with microstructure, voltage created by charge ‘soakage’ produces intracellular capacitive effects. Charge ‘soakage’ is the tendency of a capacitor to recharge itself after being discharged and the nonlinear capacitance-voltage characteristic C(V) is approximated by a linear polarization capacitance-voltage characteristic of the microstructure (dimensionless):

2 $${\rm{C}}({\rm{V}})=2{\rm{\alpha }}{\rm{V}}$$

where α > 0 is the ‘soakage’ parameter (mV^−1^) determined from the quantity of electrical charges held in the capacitance of the microstructure. It represents the capacity to hold more electrical charge or electrical energy than a linear capacitor. Inherent in the assumption of the nonlinear voltage-dependent capacitance of the microstructure is that charge ‘soakage’ reflects a density of electric charges in the microstructure irrespective if charge flow of molecular ions is from mitochondrial inactive membrane channel^32^ or the cytoskeleton. The ‘soakage’ parameter α is zero only in the absence of microstructure and so there is no voltage-independent intracellular capacitance, that is, C(V) ≠ 1 + 2αV; otherwise it is positive (α > 0). The ‘soakage’ parameter should not be confused with the ‘feedback’ parameter^33^ (β) for charge storage in the plasma membrane capacitance, which is negative (β < 0), that is C(V) = 1 + βV.

The *nonlinear* capacitor C_i_(V) = C_i_ C(V) possesses voltage-dependence at slow varying electric fields (e.g., under quasi-electrostatic conditions) giving the capacity to hold more electric charge than a linear capacitor, which can result in non-dissipative electrical signaling. The polarized macromolecules attract bound charge densities within the microstructure which are stored in the nonlinear capacitor providing a physical basis of C(V). We can glean a quantitative understanding of C(V) which can be extracted from experimental data^34, 35^. For example, if a quadratic capacitance-voltage characteristic C(V) = 2αV^2^ is used instead of (2) then a ‘kink’ shaped soliton solution (i.e., waveform) results upon solving (1); see ref. 20. Therefore C(V) is the main source of nonlinearity responsible for both shape and stability of the electrotonic signal.

The capacitance in an electrostatic system represents a way to hold electric charges. Most nonlinear capacitors are defined as C_i_(V) = dQ/dV and upon integration yields the charge-voltage relationship:

$${\rm{Q}}={{\rm{C}}}_{{\rm{i}}}\,{\rm{C}}({\rm{V}}){\rm{V}}$$

where Q is the electrical charge of the microstructure per unit length of cable (C/cm) and C_i_ is the linear voltage-independent longitudinal capacitance (F/cm). Also ∂Q/∂t = C_i_ ∂{C(V)V}/∂t is the polarizing current underlying the charge ‘soakage’. If α = 0 then no charge is stored due to the absence of microstructure. However, the contribution of microstructure to the generation of the voltage-dependent charge transfer excludes the changes in capacitance reflected through electrocompressive forces of the plasma membrane where the voltage-dependent charge transfer in the squid axon dQ/dV follows a quadratic relationship^34^. Given that the nonlinear capacitance derived from electrostriction is less than 1% of the total charge in squid axons^35^, and endogenous structures are active during the linear phase, therefore C(V) is linearly proportional to V; see e.g. ref. 32. In general, however, quadratic nonlinearity in C(V) is linked to electrocompressive forces^33^ and not the result of charge ‘soakage’ in the microstructure.

Equation (1) represents a phenomenological description of nonlinear electrostatic diffusion of charges (conduction of electric current) in a cable reduced from Maxwell’s equations^19^. When κ = 0 implies no charge ‘soakage’ is stored due to the absence of microstructure and (1) reduces to a generalized linear cable equation; compare with refs 19, 29, 36. If γ = 0 and κ = 0 then (1) reduces to electrotonic conduction of current in a “lossy” cable^13^. Finally it can be shown that the dispersion relation (see Supplementary data) is complex, and therefore the nonlinear cable equation without polarization current is dispersionless and dissipative as evident when T → ∞, so that the voltage pulse dissipates as it propagates. However, in our model, the electrotonic signals are self-generating due to the charge ‘soakage’ and they dissipate only in the absence of microstructure. This reflects upon the self-excitable nature of the model as opposed to a non-quiescent model^28^ where solitary waves are observed due to excess charge on top of plasma membrane ionic channel proteins at discrete localizations.

Given that the parameters α and κ both require quantifying, we can alleviate this problem by expressing the normalized membrane potential in non-dimensional terms via U → ακV and therefore (1) becomes a third-order non-evolutionary equation:

3 $${\rm{U}}+\partial {\rm{U}}/\partial {\rm{T}}={\partial }^{2}{\rm{U}}/\partial {{\rm{X}}}^{2}+{\rm{\gamma }}{\partial }^{3}{\rm{U}}/\partial {\rm{T}}\partial {{\rm{X}}}^{2}+2\,\partial {{\rm{U}}}^{2}/\partial {\rm{T}},\,0 < {\rm{X}} < {\rm{L}}$$

This equation is combined with a variety of boundary conditions at X = 0 and X = L (see Table 1). The only parameter γ = 0.001 is known from cable theory^19, 20^. The non-dimensionalization of the membrane potential has clear advantages that allows for the electrotonic signals to be enhanced since under normal experimental situations they are rarely encountered due to their small amplitude, possibly buried in noise during electrophysiological recordings. Equation (2) expressed in non-dimensional membrane potential leaves the dissipation term small, while the charge ‘soakage’ term which is the only nonlinear term that is not small. Given that both α and κ are small parameters, the peak amplitude of the dimensional voltage V can be found from the inverse product (1/ακ). Although α and κ are removed through the normalization process, experimentally observed electrotonic signals of around V = 5 mV; see ref. 21, can be a way to crudely estimate α for any given κ.

**Table 1:** Method of images solution coefficients for a finite neuronal branchlet.

Type of Boundary Condition	α_n_	β_n_
U_X_(0, T) = U_X_(L, T) = 0	1	1
U(0, T) = U_X_(L, T) = 0	(−1)^*n*^	−(−1)^*n*^
U_X_(0, T) = U(L, T) = 0	(−1)^*n*^	(−1)^*n*^
U(0, T) = U(L, T) = 0	1	−1

## Results

### Approximate traveling wave solutions in free space using the tanh-function expansion method

Traveling wave solutions assume a constant conduction velocity that relies on a Galilean transformation of the independent variables which reduces (3) to an ordinary differential equation: ξ = X − X_p1_ − νT where X_p_ is the initial location of the electrotonic signal positioned along the cable and ν is the velocity of the electrotonic signal moving towards ξ → ∞. The solitary wave moving in the other direction ξ → − ∞, we would use ξ = X − X_p2_ + νT. For convenience, we use the solitary wave ansatz U*(X, T) = Ω(ξ) where U* is the free space version of U on an infinite interval (−∞, ∞), with the following identities:

4 $$\begin{array}{l}\,\,\,\,\,\partial {{\rm{U}}}^{\ast }/\partial {\rm{T}}=-{\rm{\nu }}{\rm{d}}{\rm{\Omega }}/{\rm{d}}{\rm{\xi }},\,{\partial }^{2}{{\rm{U}}}^{\ast }/\partial {{\rm{X}}}^{2}={{\rm{d}}}^{2}{\rm{\Omega }}/{{\rm{d}}{\rm{\xi }}}^{2},\,{\partial }^{3}{{\rm{U}}}^{\ast }/\partial {\rm{T}}\partial {{\rm{X}}}^{2}=-{\rm{\nu }}\,{{\rm{d}}}^{3}{\rm{\Omega }}/{{\rm{d}}{\rm{\xi }}}^{3}\,{\rm{and}}\\ \partial [{{\rm{U}}}^{\ast 2}]/\partial {\rm{T}}=-{\rm{\nu }}\,{\rm{d}}[{{\rm{\Omega }}}^{2}]/{\rm{d}}{\rm{\xi }}=-2{\rm{\nu }}{\rm{\Omega }}\,({\rm{d}}{\rm{\Omega }}/{\rm{d}}{\rm{\xi }})\end{array}$$

Substitution of (4) into (3) yields

5 $${\rm{\nu }}\,{{\rm{\gamma }}{\rm{d}}}^{3}{\rm{\Omega }}/{{\rm{d}}{\rm{\xi }}}^{3}-{{\rm{d}}}^{2}{\rm{\Omega }}/{{\rm{d}}{\rm{\xi }}}^{2}+{\rm{\nu }}\,(4{\rm{\Omega }}-1)({\rm{d}}{\rm{\Omega }}/{\rm{d}}{\rm{\xi }})+{\rm{\Omega }}=0$$

with the boundary conditions for electrotonic signals Ω(±∞) = 0.

The solution of (5) can be found numerically formulated as a two-point boundary value problem. Methods for numerical integration, such as shooting methods, can be used or alternatively a direct method can also be used to analytically solve (5). This has quantitative improvements over other methods that employ traveling wave fronts or phase-plane methods that require some numerical computation to estimate the velocity of the wave (e.g. shooting method); see e.g., ref. 37. One such direct method is the so-called tanh-function expansion method; see e.g., ref. 38, where a new independent variable is introduced:

$${\rm{y}}=\,\tanh \,({\rm{\xi }})$$

with

$$\begin{array}{rcl}{\rm{d}}{\rm{\Omega }}/{\rm{d}}{\rm{\xi }} & = & [({\rm{1}}-{{\rm{y}}}^{2}){\rm{d}}f/{\rm{dy}}]\\ {{\rm{d}}}^{2}{\rm{\Omega }}/{{\rm{d}}{\rm{\xi }}}^{2} & = & [-2{\rm{y}}(1-{{\rm{y}}}^{2}){\rm{d}}f/{\rm{dy}}+{(1-{{\rm{y}}}^{2})}^{2}\,{{\rm{d}}}^{2}f/{{\rm{dy}}}^{2}]\\ {{\rm{d}}}^{3}{\rm{\Omega }}/{{\rm{d}}{\rm{\xi }}}^{3} & = & [2(1-{{\rm{y}}}^{2})(3{{\rm{y}}}^{2}-1)\,{\rm{d}}f/{\rm{dy}}-6{\rm{y}}{(1-{{\rm{y}}}^{2})}^{2}{{\rm{d}}}^{2}f/{{\rm{dy}}}^{2}+{(1-{{\rm{y}}}^{2})}^{3}{{\rm{d}}}^{3}f/{{\rm{dy}}}^{3}]\end{array}$$

where Ω(ξ) → ƒ(y) and ƒ(±1) → 0.

Substituting the above new variables into (5) results in the following expression:

7 $$\begin{array}{rcl}f-{\rm{\nu }}(1-{{\rm{y}}}^{2})\,{\rm{d}}f/{\rm{dy}} & = & [-2{\rm{y}}(1-{{\rm{y}}}^{2})\,{\rm{d}}f/{\rm{dy}}+{(1-{{\rm{y}}}^{2})}^{2}\,{{\rm{d}}}^{2}f/{{\rm{dy}}}^{2}]\\  &  & -{\rm{\gamma }}{\rm{\nu }}[2(1-{{\rm{y}}}^{2})(3{{\rm{y}}}^{2}-1){\rm{d}}f/{\rm{dy}}-6{\rm{y}}{(1-{{\rm{y}}}^{2})}^{2}{{\rm{d}}}^{2}f/{{\rm{dy}}}^{2}\\  &  & +{(1-{{\rm{y}}}^{2})}^{3}{{\rm{d}}}^{3}f/{{\rm{dy}}}^{3}]-4{\rm{\nu }}f(1-{{\rm{y}}}^{2}){\rm{d}}f/{\rm{dy}}\end{array}$$

Note: For a variable width of the solitary wave, a more generalized tanh-function expansion method can be used with y = tanh (μξ) where μ donotes the width of the solitary wave^38^.

The tanh-function expansion method admits the use of a *finite* expansion of the form ƒ(y) = ∑a_n_y^n^ where n is a positive integer to be determined by equating the powers of y in the resultant equation upon its substitution into (7). To determine the parameter n, we balance the highest order linear terms with the highest order nonlinear terms, which gives n = 2. Therefore the solution takes the form:

8 $$f({\rm{y}})={{\rm{a}}}_{{\rm{o}}}+{{\rm{a}}}_{{\rm{o}}}({{\rm{a}}}_{{\rm{1}}}-1)\,{\rm{y}}-{{\rm{a}}}_{{\rm{1}}}{{\rm{a}}}_{{\rm{o}}}\,{{\rm{y}}}^{2}$$

Unlike a perturbation expansion, (8) represents a finite expansion and so no higher-order terms are required. As a result, there is no test of convergence that is required to be undertaken as expected when using perturbation methods. If y →  −1 then from (8) and the boundary condition ƒ(−1) → 0 yields a_1_ = 1 and the solution takes the form:

9 $$f({\rm{y}})={{\rm{a}}}_{{\rm{o}}}({\rm{1}}-{{\rm{y}}}^{2})$$

If y → 1 then from (9) upon substituting into (7) and the boundary condition ƒ(1) → 0 yields the velocity of the wave:

$${\rm{\nu }}=(3/2)[1/(1-4{\rm{\gamma }})]$$

Substitution of y = tanh(ξ) into (9) yields the traveling wave solution for a solitary wave of unitary width and moving at speed ν :

10 $${{\rm{U}}}^{\ast }({\rm{X}},{{\rm{X}}}_{{\rm{p}}};{\rm{T}})={{\rm{a}}}_{{\rm{o}}}\,{\rm{sech}}^{{\rm{2}}}({\rm{X}}-{{\rm{X}}}_{{\rm{p}}}-\nu {\rm{T}})$$

where a_o_ is the dimensionless amplitude determined to be a_o_ ≈ 3γ + 3/(4ν) (see Supplementary data). Therefore the amplitude of the solitary wave decreases with distance from the initial position. Substituting the velocity ν into the dimensionless form of the traveling wave solution is given as

11 $${{\rm{U}}}^{\ast }({\rm{X}},{{\rm{X}}}_{{\rm{p}}};{\rm{T}})\approx (3/8)[2-1/\nu ]{{\rm{s}}{\rm{e}}{\rm{c}}{\rm{h}}}^{2}({\rm{X}}-{{\rm{X}}}_{{\rm{p}}}-\nu {\rm{T}})$$

The approximate traveling wave solution governed by (11) is known as a solitary-wave solution (or quasi-soliton) corresponding to an electrotonic signal propagating at a constant speed ν > 0.5. The solitary-wave solution is only an approximate solution of (3) that is shown to be stable based on local stability analysis (see Supplementary data).

**Figure 4:**
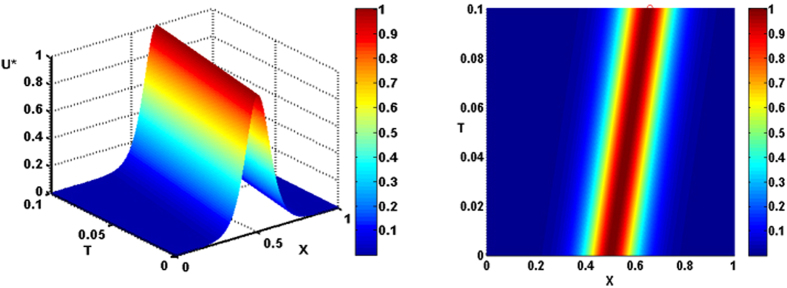
A propagating quasi-soliton expressed in terms of spatiotemporal evolution of normalized membrane potential U*(X, X_P_; T)/U*(X_P_ X_P_; 0) as a function of electronic distance (X) and dimensionless time (T) along an infinitely long neuronal branchlet obtained from (11). Parameters used were: ν = 1.506, γ = 0.001 and X_p_ = 0.5.

The results presented in Fig. 4 show a quasi-soliton as the spatiotemporal evolution of the normalized membrane potential U*(X, X_p_;T)/U*(X_p_, X_p_;0) in non-dimensional terms along an infinite cable (in free-space) implemented in *Matlab* software package. The quasi-solitons are insensitive to the initial location of their positioning X_p_ as the hyperbolic secant function reaches a maximum value of unity when X = X_p_. These solitary waves are not chaotic since they exhibit globally regular amplitudes and a constant velocity of propagation. The results are presented for a spatially homogeneous medium where the solitary waves propagate with a constant velocity and amplitude that is independent of their initial position. This quasi-soliton possesses no energy loss due to charge ‘soakage’ as charge keeps coming out of the capacitor of the polarized microstructure, which is absent in energy consuming action potentials. As shown in Fig. 4 (right-hand-side), the velocity of the quasi-soliton is inversely proportional to the slope of this graph. As can be seen the co-ordinate for the first and last points are (X_1_, T_1_) = (0.5, 0) and (X_2_, T_2_) = (0.6506, 0.1), respectively. Thus slope = (T_2_ − T_1_)/(X_2_ − X_1_) = (0.1 − 0)/(0.6506 − 0.5) = 0.6641 and the dimensionless velocity is inversely proportional to this slope 1/0.6641 ≈1.506.

### Solitonic properties

The solitonic property of a solitary wave (or quasi-soliton) is that asymptotically it preserves its shape and velocity on collision with other quasi-solitons^39^. The linear superposition of electrotonic signals is assumed to approximate the collision:

12 $${{\rm{U}}}^{\ast }({\rm{X}},{{\rm{X}}}_{{\rm{p}}1}+{{\rm{X}}}_{{\rm{p}}2};{\rm{T}})\approx (3/8)[2-1/\nu ]\{{{\rm{s}}{\rm{e}}{\rm{c}}{\rm{h}}}^{2}({\rm{X}}-{{\rm{X}}}_{{\rm{p}}1}-\nu {\rm{T}})+({{\rm{s}}{\rm{e}}{\rm{c}}{\rm{h}}}^{2}({\rm{X}}-{{\rm{X}}}_{{\rm{p}}2}+\nu {\rm{T}})\}$$

**Figure 5:**
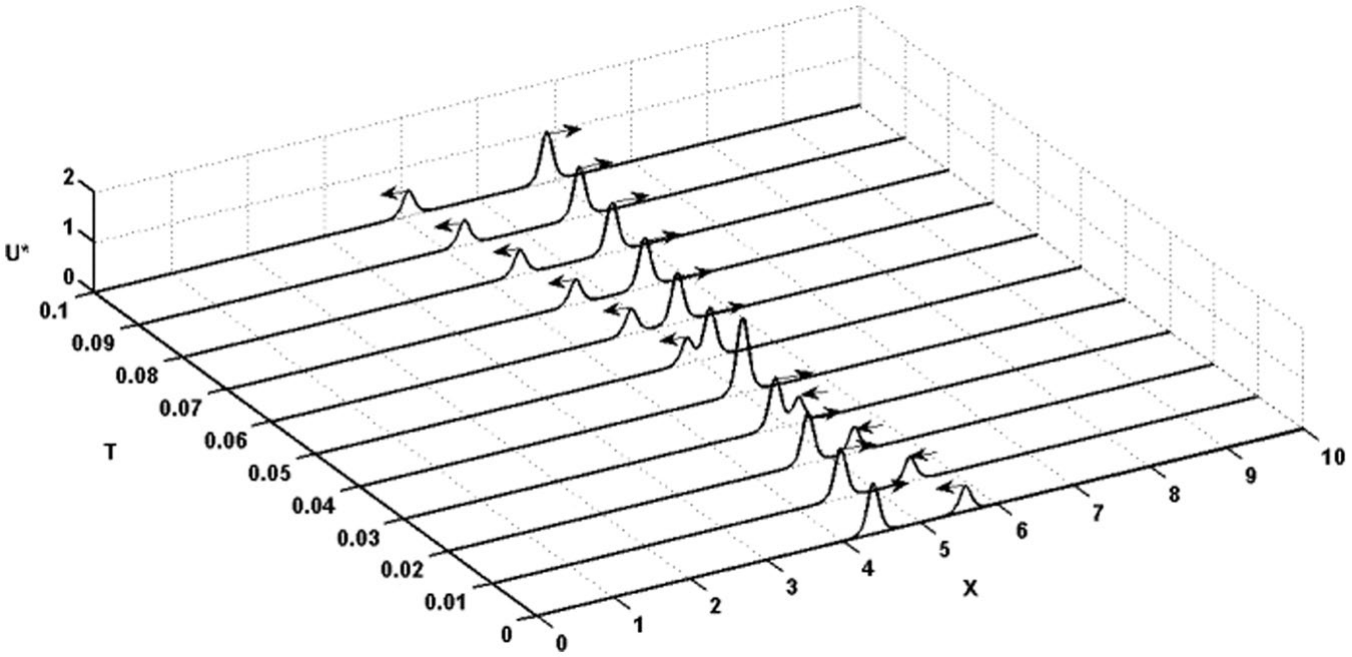
Two oppositely directed quasi-solitons (solitary waves) along an infinitely long neuronal branchlet result in a head-on collision. The collision occurred by linear superposition of the solitary waves. To differentiate the amplitudes of the traveling wave solutions expressed in terms of the membrane potential U*(X, X_p__1_ + X_p__2_; T) obtained from (12) as a function of electrotonic distance (X) and dimensionless time (T), the quasi-soliton moving to the right was normalized by U*(X_p__1_, X_p__1_ + X_p__2_; 0) and the quasi-soliton moving to the left was half-normalized by 2U*(X_p__2_, X_p__1_ + X_p__2_; 0). Both quasi-solitons propagate with a dimensionless conduction velocity of ν = 1.506. Parameters used were: γ = 0.001, X_p__1_ = 4.398, X_p__2_ = 5.602.

We illustrate the collision between two quasi-solitons with identical velocities in order to test their responses after collision. The electrotonic signal has fixed velocity and is not amplitude-dependent. The result of the head-on collision of two quasi-solitons is simulated resulting in an elastic interaction with electrotonic signals remaining unchanged after collision. The simulated result for the two quasi-solitons (solitary waves) approaching each other with the one moving to the right having a normalized amplitude of U*(X_p__1_, X_p__1_ + X_p__2_; 0) and the other moving to the left having a half-normalized amplitude of 2U*(X_p__2_, X_p__1_ + X_p__2_; 0) is shown in Fig. 5. The elastic interaction of quasi-solitons stems from the absence of recovery processes known to be the major cause of collapse of colliding spikes^4^. The elastic interaction between two quasi-solitons preserves their shape, amplitudes and velocities. Velocities are parameter-dependent on the ratio of the Maxwell’s time-constant and the membrane time-constant. The dynamics of electrotonic signals are shown to interact during collision, although no annihilation of electrotonic signals due to conservation of energy. The time of collision occurs at T = (X_p2_ − X_p1_)/2ν which is 0.04 with X_p1_ = 0.5–4ν/100 = 0.4398 and X_p2_ = 0.5 + 4ν/100 = 0.5602 as shown in Fig. 5.

**Figure 6:**
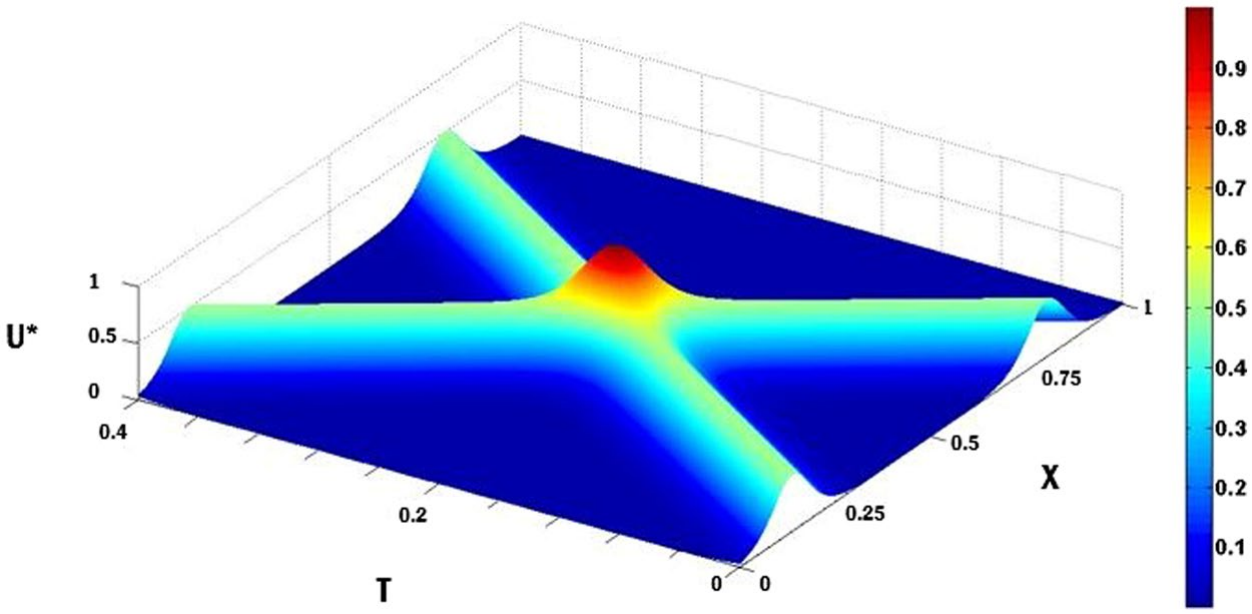
An elastic interaction between two oppositely directed quasi-solitons after a head-on collision along an infinitely long neuronal branchlet. Traveling wave solution in terms of a normalized membrane potential U*(X, X_p1_ + X_p2_; T)/(1 + 2γ) obtained from (12) as a function of electrotonic distance (X) and dimensionless time (T). Both quasi-solitons propagate with a dimensionless conduction velocity of ν = 1.506. The interaction between quasi-solitons occurred by linear superposition of quasi-solitons. Parameters used were: γ = 0.001, X_p__1_ = 0.15, X_p__2_ = 0.85.

If the collision between two solitary waves is elastic (i.e. preserves their shape and velocities) then it can be described as being solitonic. This is the major property of soliton as a self-reinforcing solitary wave. In a non-dispersive medium, solitons are dissipative, but only in the sense that in the presence friction, they gradually decelerate and become smaller and they will eventually decay as T → ∞, but only when dissipative energy is not fueled by the reservoir of electrical charges held by the intracellular capacitance and which in turn releases stored energy for maintenance of the electrotonic signal. In a linear dissipative medium the dynamics of soliton collisions appear to be absorbing one another, i.e., solitons pass through one another^40^. This is shown more clearly in Fig. 6 where two quasi-solitons undergo linear superposition at collision. The time of collision occurs at T = (X_p2_ − X_p1_)/2ν which is 0.234 (see Fig. 6). The simulation shows that the two electrotonic signals pass through one another and are not deformed after collision, preserving their shape, amplitude and velocity. More importantly, after the interaction they continue to propagate without dissipating, thus providing unequivocal support for quasi-solitons to be solitons. The defining condition for solitons is that they pass through one another and not merely reflect from one another. The simulation shows the existence of a point where there is only a single peak, suggesting that the solitons absorb one another during the collision. Also the traveling wave solution U*(X, T) = Ω(ξ) can be shown to satisfy the following conditions^38^: Ω′(ξ) = Ω″(ξ) = Ω′″(ξ) = 0 where prime denotes differentiation with respect to ξ, further reinforcing the electrotonic signals as solitons.

### The effect of boundaries

The method of images technique^30, 31^ can be employed to determine the appropriate spatiotemporal evolution of membrane potential on a finite domain for small values of time. On a finite interval, the effect of boundaries causes reflections to occur, which must be added to the original free space solitary wave U*(X, X_p_;T) solution. Therefore, the solitary wave in a finite cable of electrotonic length L is expressed by

13 $${\rm{U}}({\rm{X}},{\rm{T}}\to 0)=\sum _{{\rm{n}}=-\infty }^{\infty }\{{{\rm{\alpha }}}_{{\rm{n}}}{{\rm{U}}}^{\ast }({\rm{X}},2{\rm{nL}}-{{\rm{X}}}_{{\rm{p}}};{\rm{T}}\to 0)+{{\rm{\beta }}}_{{\rm{n}}}{{\rm{U}}}^{\ast }({\rm{X}},2{\rm{nL}}+{{\rm{X}}}_{{\rm{p}}};{\rm{T}}\to 0)\}$$

where α_n_ and β_n_ depend on boundary conditions and are given in Table 1.

The boundary condition U_X_ = 0 characterizes a reflecting boundary, where the conduction of current is reflected often called a “sealed-end” boundary condition. The boundary condition U = 0 represents an absorbing boundary, where the conduction of current is grounded to its resting state (or voltage –clamped if not zero) often called “killed-end” or “short-circuit” boundary condition. Additional boundary conditions not included in Table 1 are a combination of the two denoted as a “leaky-end” boundary condition where conduction of current is transmitted through a resistance representing a gap-junction^41^. Another common boundary condition is the “natural” boundary condition where a time derivative U_T_ is used in addition to the “leaky–end” reflecting an isopotential structure expressed electrically in terms of a RC circuit that is added to the cable at both ends, resulting in a dumbbell cable^42^. Both “leaky-end” and “natural” boundary conditions introduce an additional term in (13) upon use of the method of images technique^43^.

**Figure 7:**
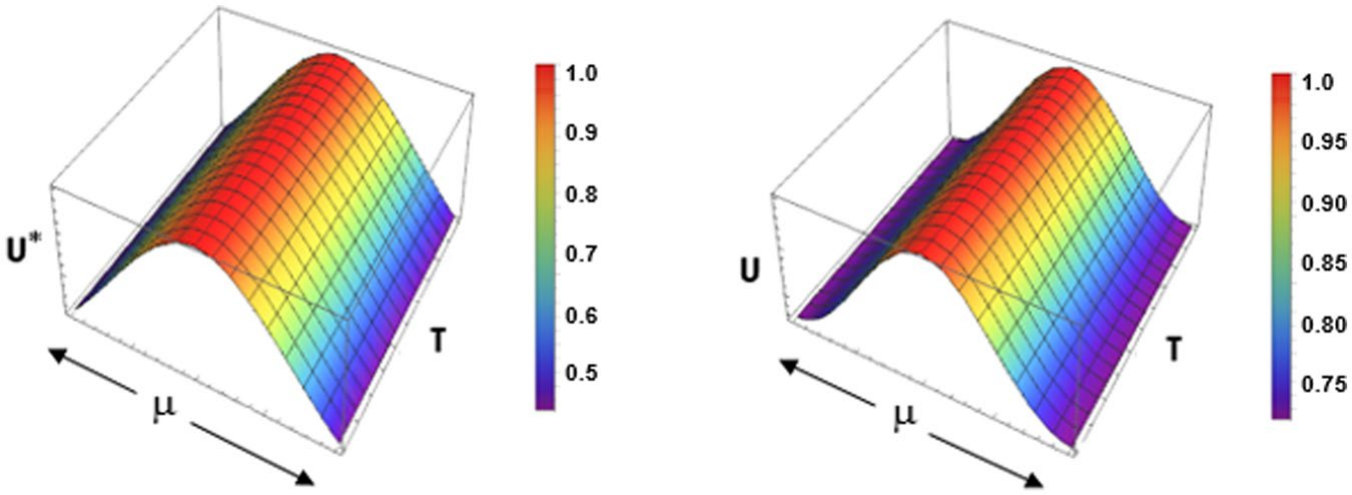
The effect of sealed-end boundary conditions on the quasi-soliton profile showing the apex with a variable width. A generalized tanh-function expansion method with y = tanh (μξ) where μ donotes the width of the profile as a normalized Gaussian curve. The spatiotemporal evolution of the normalized membrane potential U(X_P_ , T)/U(X_P_ , 0) in non-dimensional terms obtained from (13) with n = 0 (left-hand side) and n = ±10 (right-hand side).

The simulations with finite cables are indistinguishable from the free space solutions. One reason is that the zero term dominates the expansion in (13), which governs the free space solution at small times. Therefore no damping of the electrotonic signals occurs due to the effects of the boundaries. The marginal differences in the normalized Gaussian profile can be seen if a more generalized tanh-function expansion method is employed^38^. As indicated in Fig. 7 the normalized Gaussian profile appears to have a lower variance in the curved profile compared to the free space solution, which is attributed to the reflection of non-zero terms from the sealed-ends boundary conditions.

## Discussion

A model of a neuronal branchlet that included a phenomenological description of polarized microstructure in terms of intracellular capacitive effects was shown to be capable of influencing subcellular electrical signal propagation under quasi-electrostatic conditions (slow moving electric field). The resultant intracellular capacitive effect of polarized microstructure gives rise to self-excitability due to charge ‘soakage’ held in voltage-dependent capacitance of the microstructure invoking traveling wave pulses instead of traveling wave fonts, as one would expect without any recovery processes inherent in the model.

These traveling wave pulses were shown to be conducted through a novel mode of conduction exclusively for branchlets with microstructure. The stable electrotonic signals propagated due to stored energy in the microstructure. The crucial test for solitary waves to be electrical solitons is robustness to collision. Electrical solitons do not undergo nonlinear amplitude modulation during collision because linear superposition of solitary waves is assumed in a linear dissipative medium^40^. Based on linear superposition simulating interaction, the tanh-function expansion method enabled to directly procure approximate traveling wave solutions (quasi-solitons); their collision dynamics remained unchanged as they passed through one another, providing support for solitary waves to be electrical solitons. To the best of our knowledge, our model provides a first attempt at describing electrotonic signals denoting the direct spread of current in branchlets by means of solitonic conduction based on cable theory as opposed to transmission line theory (electrical lattices)^40^ or reaction-diffusion theory^10, 11^.

Traveling waves conducted as stereotypical action potentials are energy consuming^44^. Therefore action potentials dissipate with time and annihilate upon collision^4^. Electrical solitons dissipate, but because of microstructure and the resultant distribution of charge ‘soakage’, the dissipative energy was shown to be conserved. Furthermore, electrical solitons did not annihilate upon collision because unlike action potentials with the refractory period, the electrotonic signals are maintained by the flux associated with the polarization current flowing through the miscrostructure. Therefore, the model possess attributes like stability and elastic interaction upon head-on collision that differ significantly from stereotypical action potentials attributed to the gating currents through the plasma membrane, including unstable pulses serving as threshold conditions for igniting these stereotypical action potentials^45^.

What might be the function of electrotonic signals propagating along fine distal branchlets? Electrotonic signals without recovery are suitable in transmitting information through physical interaction of electrical charges held by microstructure and not synaptic inputs^46^. The stable non-decremented electrotonic signals originating in branchlets could play a functional role in heterosynaptic plasticity. Also electrical solitons conserve energy and so they can decode local information permanently. The stable dynamics of electrotonic signals provide a mechanistic explanation to retrieve long-term memories^47^. Our view is that backpropagating action potentials^48^ are unencumbered by solitonic conduction for the reason that they become erratic and unpredictable prone to propagation failures at diameters smaller than 0.5 μm^49^. Based on the permanence of these electrical solitons, it is unlikely that backpropagating action potentials are involved in higher brain functions such as in conceptual tasks. While for those action potentials that are initiated in thin dendrites (see ref. 50 for a review) we propose two specialized interdependent signals in local information processing: (1) dendritic spikes for encoding/imprinting information and (2) solitons for decoding/retrieval information in distal most dendrites of cortical neurons.

The function of solitonic conduction in branchlets may differ in thin axons and thin dendrities. Plasma membrane ion channel noise causes action potential reliability to be significantly diminished in their information carrying capacity in thin axons (<0.5 μm diameter)^51^. The lower limit to axon diameter is about 80 nm^52^. Experiments revealed very thin pyramidal cell axons can conduct action potentials reliably^53^ and simulations show that action potentials in very thin axons are unlikely to fail to propagate due to ion channel noise^51^. This suggests conduction of action potentials in thin axons is not localized in the axonal branchlets. If neurons are designed for *presynaptic information processing* of local information to thin dendritic branchlets and not available at the site of spike initiation then electrotonic potentials can be locally integrated within the branchlets and then integrated again at one or more main intrinsic action potential initiation zones^54, 55^.

Our view, however, is that in the presence of microstructure, solitons in thin distal dendrites are the agglomeration of electrical charge densities arising as a result of electrotonic signal interactions. If the non-stereotypical spike is a composite of soliton interactions then the propagation of non-stereotypical spikes could carry dynamically-rich information generated by propagation of localized soliton resonant interactions that occur and influence the generation of ionic flow in neuronal branchlets. The presence of localized electrotonic signals reinforces preferentially local integration in different parts of the neuron and can lead to meaningful electrotonic information processing (see for example ref. 56). To test our hypothesis of solitonic conduction in branchlets designed exclusively for local electrotonic information processing, the minute intracellular volumes that go beyond the limiting spatial resolution of confocal microscopes and two-photon laser scanning fluorescence microscopy^57^ will need to be investigated with new technology that would enable to record and image the electrotonic signals in fine distal branchlets of neurons at higher spatial resolutions using dielectric scanning microscopy on neurons^58, 59^.

In their experimental study, Gonzalez-Perez and colleagues^21^ observed pulses under 10 mV in amplitude that did not annihilate upon collision. Although their reference to solitons as ‘action potentials’ leads to confusion they further resorted to an electromechanical explanation without a clear indication as to why a purely electrical phenomenon should be re-packaged in terms of electromechanical pulses. Recently, El Hady and Machta^60^ suggested that mechanical surface waves may be caused by charge separation leading to changes in capacitance due to compressive forces on the membrane (electrostriction), yet it is known that the nonlinear capacitance derived from electrostriction is expected to contribute less than 1% of the total capacitance^35^. Therefore, any electromechanical traveling waves would be negligible. In accordance with the result of Gonzalez-Perez and colleagues^21^, our model supports their experimental findings without considering adiabatic phenomena during a nerve pulse, but through charge reservoirs within the neuronal microstructure, thus reconciling the difference between electrophysiological models and thermodynamic postulates without the need for postulating mechanical soliton models.

## Electronic supplementary material

Appendix A
