# Erratum: Solitonic conduction of electrotonic signals in neuronal branchlets with polarized microstructure

**DOI:** 10.1038/s41598-017-07626-6

**Published:** 2017-09-06

**Authors:** R. R. Poznanski, L. A. Cacha, Y. M. S. Al-Wesabi, J. Ali, M. Bahadoran, P. P. Yupapin, J. Yunus

**Affiliations:** 1 Faculty of Biosciences and Medical Engineering, Universiti Teknologi Malaysia, 81310 Johor Bahru, Malaysia; 2 Laser Centre, IBNU SINA ISIR, Universiti Teknologi Malaysia, 81310 Johor Bahru, Malaysia; 3 Faculty of Science, Universiti Teknologi Malaysia, 81310 Johor Bahru, Malaysia; 4 Department of Physics, Shiraz University of Technology, Shiraz, 313-71555 Iran; 5 Department for Management of Science and Technology Development, Ton Duc Thang University, Ho Chi Minh City, District 7 Vietnam; 6 Faculty of Electrical & Electronics Engineering, Ton Duc Thang University, Ho Chi Minh City, District 7 Vietnam

*Scientific Reports* **7**:2746; doi:10.1038/s41598-017-01849-3; Article published online 31 May 2017

The incorrect version of the Supplementary Information was inadvertently published previously. This has now been corrected; the PDF version of the paper was correct from the time of publication.

