# Supplementary material for: Solitonic conduction of electrotonic signals in neuronal branchlets with polarized microstructure
Source: Sci Rep. 2017 May 31;7:2746. doi: 10.1038/s41598-017-01849-3 (PMC5451471; doi:10.1038/s41598-017-01849-3)
Supplement: Supplementary file 1 — Appendix A [file 41598_2017_1849_MOESM1_ESM.docx]

**Solitonic conduction of electrotonic signals in neuronal branchlets with polarized microstructure**

R.R.Poznanski, ^1,*^, L.A. Cacha^2^, Y.M.S. Al-Wesabi^1^, J. Ali^2,3^, M. Bahadoran^4^, P.P.Yupapin^5, 6^, and J.Yunus^1^

^1^Faculty of Biosciences and Medical Engineering, Universiti Teknologi Malaysia 81310 Johor Bahru, Johor, Malaysia

^2^Laser Centre, IBNU SINA ISIR, Universiti Teknologi Malaysia 81310 Johor Bahru, Johor, Malaysia

^3^Faculty of Science, Universiti Teknologi Malaysia, 81310 Johor Bahru, Johor, Malaysia

^4^Department of Physics, Shiraz University of Technology, Department Shiraz 313-71555, Iran

^5^Department for Management of Science and Technology Development, Ton Duc Thang University, Ho Chi Minh City, District 7, Vietnam

^6^Faculty of Electrical & Electronics Engineering, Ton Duc Thang University, Ho Chi Minh City, District 7, Vietnam

^*^Corresponding author: [poznanski@biomedical.utm.my](mailto:poznanski@biomedical.utm.my)

**APPENDIX A. Supplementary data**

**1. Dispersion relation**

Eliminating the nonlinear term $\kappa\frac{\partial[C\left( V \right)V]}{\partial T}$ from (1) yields the linearized version of the cable equation:

$V+\frac{\partial V}{\partial T}=\frac{\partial^{2}V}{\partial X^{2}}+\gamma\frac{\partial^{3}V}{\partial T\partial X^{2}}$ (A1)

The most elementary wave solution of this equation is the harmonic wave

$V\left( X,T \right)=A exp[i\left( kX+\omega T \right)]$ (A2)

where A is the amplitude of the wave, $k$ is the wave number and ω is the angular frequency. In order for V(X,T) given by (A2) to be a solution of (A1), ω and k must satisfy the relation:

$\omega=i\frac{(1+k^{2})}{1+\gamma k^{2}}$ (A3)

A “dispersion relation” is connected to the phase velocity ν_p_= ω/ $k$ and the group velocity ν_g_ = ∂ω/∂$k$ only if the problem is non-dissipative, i.e. ω must be real when$k$ is real. For this dispersion relation given by (A3), the system is dissipative, so the linearized cable equation (A1) involves energy dissipation. Substituting (A3) into (A2) gives the solution:

$V\left( X,T \right)=exp[ikX-\frac{(1+k^{2})}{1+\gamma k^{2}}T]$ (A4)

The dissipation, the exponential decay of (A4) is obviously clear for $T.$

**2. Determination of the amplitudes**

From (10) an approximate solitary traveling wave solution for the electrotonic signal with dimensionless amplitude is

$U^{*}\left( X,T \right)=a_{0}\mathrm{sech}^{2} (X-X_{p}-vT)$ (A5)

Upon differentiating (A5) we have the following derivates:

$\frac{\partial U^{*}}{\partial T}=2vU^{*}\tanh(X-X_{p}-vT)$

$\frac{\partial^{2}U^{*}}{\partial X^{2}}=4U^{*}-6\frac{U^{*2}}{a_{0}}$

$$\frac{\partial^{3}U^{*}}{\partial T\partial X^{2}}=8vU^{*}\tanh(X-X_{p}-vT)-24vU^{*2}\frac{\tanh(X-X_{p}-vT)}{a_{0}}$$

$$\frac{\partial U^{*2}}{\partial T}=4vU^{*2}\tanh(X-X_{p}-vT).$$

Substituting the above derivates into (3) in order to verify that (A5) is a solution. In case of the nontrivial solution U≠0, the following relations must hold:

$2v\tanh(X-X_{p}-vT)-3-8v\gamma\tanh\left( X-X_{p}-vT \right)=0$ (A6)

$\frac{6}{a_{0}}+24v\gamma\frac{\tanh\left( X-X_{p}-vT \right)}{a_{0}}-8v\tanh\left( X-X_{p}-vT \right)=0$ (A7)

From (A6) we have $\tanh\left( X-X_{p}-vT \right)\approx1$ for $0<T<(2-X+X_{p})/v$ and substituting this into (A7) we have

$\frac{6}{a_{0}}+36\frac{\gamma}{(1-4\gamma)a_{0}}-\frac{12}{(1-4\gamma)}\approx0$ (A8)

It can be readily shown that (A8) holds when$a_{0}\approx3\gamma+\frac{3}{4v}$.

**3. Local stability analysis**

The equilibrium solution of (3) with boundary conditions $U()=0$ is $U= 0$. Linearization of (3) yields the linearized version of the nonlinear cable equation:

$U+\frac{\partial U}{\partial T}=\frac{\partial^{2}U}{\partial X^{2}}+\gamma\frac{\partial^{3}U}{\partial T\partial X^{2}}$ (A9)

Consider the solution of this equation to take the form:

$U\left( X,T \right)= exp[\theta T+ikX]$ (A10)

where $\theta$ is the eigenvalue of the wave, $k$ is the wave number. Equation (A10) is substituted into (A9) to order to obtain the ‘dispersion’ relation:

$\theta=-\frac{(1+k^{2})}{1+\gamma k^{2}}$ (A11)

For local stability $\theta<0$ and for this dispersion relation given by (A11), the equilibrium solution is therefore shown to be locally stable for all $>0$.
